# Supplementary material for: Development and Validation of a Machine Learning Wrist-worn Step Detection Algorithm with Deployment in the UK Biobank
Source: medRxiv. 2023 Feb 22:2023.02.20.23285750. Preprint. [Version 1] doi: 10.1101/2023.02.20.23285750 (PMC10187326; doi:10.1101/2023.02.20.23285750)
Supplement: Supplement 1 [file media-1.pdf]

Supplemental Materials

Supplemental Table 1: Summary of Datasets ii

Supplemental Table 2: Step Count Results and Performance Metrics Based in the OxWalk Dataset iii

Supplemental Table 3: Internal Validation Gait Classification Metrics for the Self-Supervised Learning Model iv

Supplemental Table 4: Step Count Results and Performance Metrics Based in the External Clemson Shimmer3 Dataset Across all Participants and Gait Subtypes v

Supplemental Figure 1: A foot-facing action camera clipped at the participant's waistline recording video for step count annotation. The participant is also wearing Axivity AX3 accelerometers at the dominant wrist and at the hip. vi

Supplemental Figure 2: Flow diagram for descriptive and survival analyses in UK Biobank physical activity cohort. vii

Supplemental Figure 3: Peak step cadence in the UK Biobank Physical Activity Cohort, summarised by self-reported walking pace and sex. viii

Supplemental Figure 4: Spearman's rank correlation plot between accelerometer-based physical activity metrics, including overall acceleration, daily step count, and machine learning activity classifications of time spent in sleep, sedentary behaviour, light activity, and moderate-to-vigorous activity (MVPA). ix

Supplemental Note 1: Comparative Step Detection Models x

Supplemental Note 2: Statistical Analysis, Further Detail xi

Supplemental Note 3: Field and Code Usage xii

Supplemental Table 1: Summary of Datasets

Characteristic	OxWalk[16]	Clemson Shimmer3[19]	UK Biobank Physical Activity Cohort[4]
Institution	University of Oxford	Clemson University	UK Biobank
Number of Participants	39	30	~100,000
Sensor	Axivity AX3<br>triaxial accelerometer	Shimmer3<br>9-axis inertial measurement unit	Axivity AX3<br>triaxial accelerometer
Sampling Rate	25 Hz and 100 Hz	15 Hz	100 Hz
Body Location	Dominant wrist and dominant hip	Non-dominant wrist, hip, and ankle	Dominant wrist
Activity Protocol	Unscripted<br>free-living	Laboratory scripted sessions of regular, semiregular, and irregular gait	Unscripted<br>free-living
Measurement Window	1 hour	37 minutes	7 days
Ground Truth Capture	Foot-facing<br>video camera<br>worn at waist	Participant-facing video camera held<br>by researcher	None
Annotation of data	Timestamped steps by two annotators	Timestamped steps<br>and shifts	N/A
Label granularity	Timestamps to<br>0.001 s precision	Timestamps to<br>0.001 s precision	N/A
Participant Age	38.5 (SD 14.0) years<br>Range: 19 - 81	21.9 (SD 2.4) years	62.4 (SD 7.8) years<br>Range 43 - 79
Participant Sex	19 Female,<br>20 Male	15 Female,<br>15 Male	56% Female, 44% Male

Supplemental Table 2: Step Count Results and Performance Metrics Based in the OxWalk Dataset

	Ground Truth	Acceleration Threshold Model[8]	Verisense Algorithm[25]	SSL Hybrid Model
Total Steps	62,782	106,160	58,356	62,025
Mean Steps	1,613	2,722	1,496	1,590
Mean Bias (%)		69.1%	-7.2%	-1.3%
MAPE (SD)		231.3% (254.0%)	63.5% (103.6%)	12.5% (15.5%)

MAPE (SD): Mean Absolute percent error (standard deviation)

Supplemental Table 3: Internal Validation Gait Classification Metrics for the Self-Supervised Learning Model

	F1	Cohen's Kappa	Precision	Recall	Accuracy
SSL Hybrid Model	0.842 ± 0.148	0.7890 ± 0.149	0.908 ± 0.088	0.807 ± 0.192	0.948 ± 0.047

Supplemental Table 4: Step Count Results and Per-Participant Performance Metrics Based in the External Clemson Shimmer3 Dataset

**Overall Per-Participant**	Ground Truth	Acceleration Threshold[8]	Verisense[25]	SSL Hybrid Model
Total Steps	54,387	79,885	44,813	45,357
Mean Participant Steps	1,813	2,663	1,494	1,512
% Mean Bias		+46.9%	-17.6%	-16.6%
MAPE (SD)		47.5% (11.5%)	17.3% (10.2%)	16.5% (8.8%)
**Regular Gait**				
Total Steps	31,401	29,993	26,155	28,433
Mean Steps per Session	1,047	1000	872	948
% Mean Bias		-4.5%	-16.8%	-8.5%
MAPE (SD)		6.4% (8.1%)	16.3% (11.4%)	9.2% (9.5%)
**Semiregular Gait**				
Total Steps	18,444	24,488	14,033	13,803
Mean Steps per Session	615	816	468	615
% Mean Bias		32.8%	-23.9%	-25.2%
MAPE (SD)		33.5% (12.6%)	23.9% (11.5%)	25.8% (13.8%)
**Irregular Gait**				
Total Steps	4,542	25,404	4,625	3,121
Mean Steps per Session	151	847	154	104
% Mean Bias		459.3%	+1.8%	-31.3%
MAPE (SD)		464.0% (84.3%)	17.3% (14.1%)	31.6% (15.9%)

MAPE (SD): Mean absolute percent error (standard deviation)

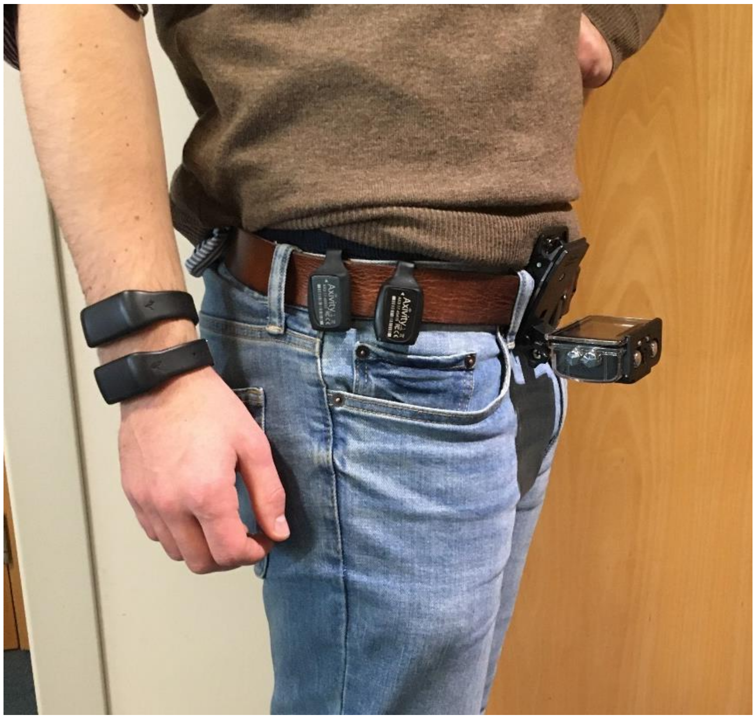

Supplemental Figure 1: A foot-facing action camera clipped at the participant’s waistline recording video for step count annotation. The participant is also wearing Axivity AX3 accelerometers at the dominant wrist and at the hip.

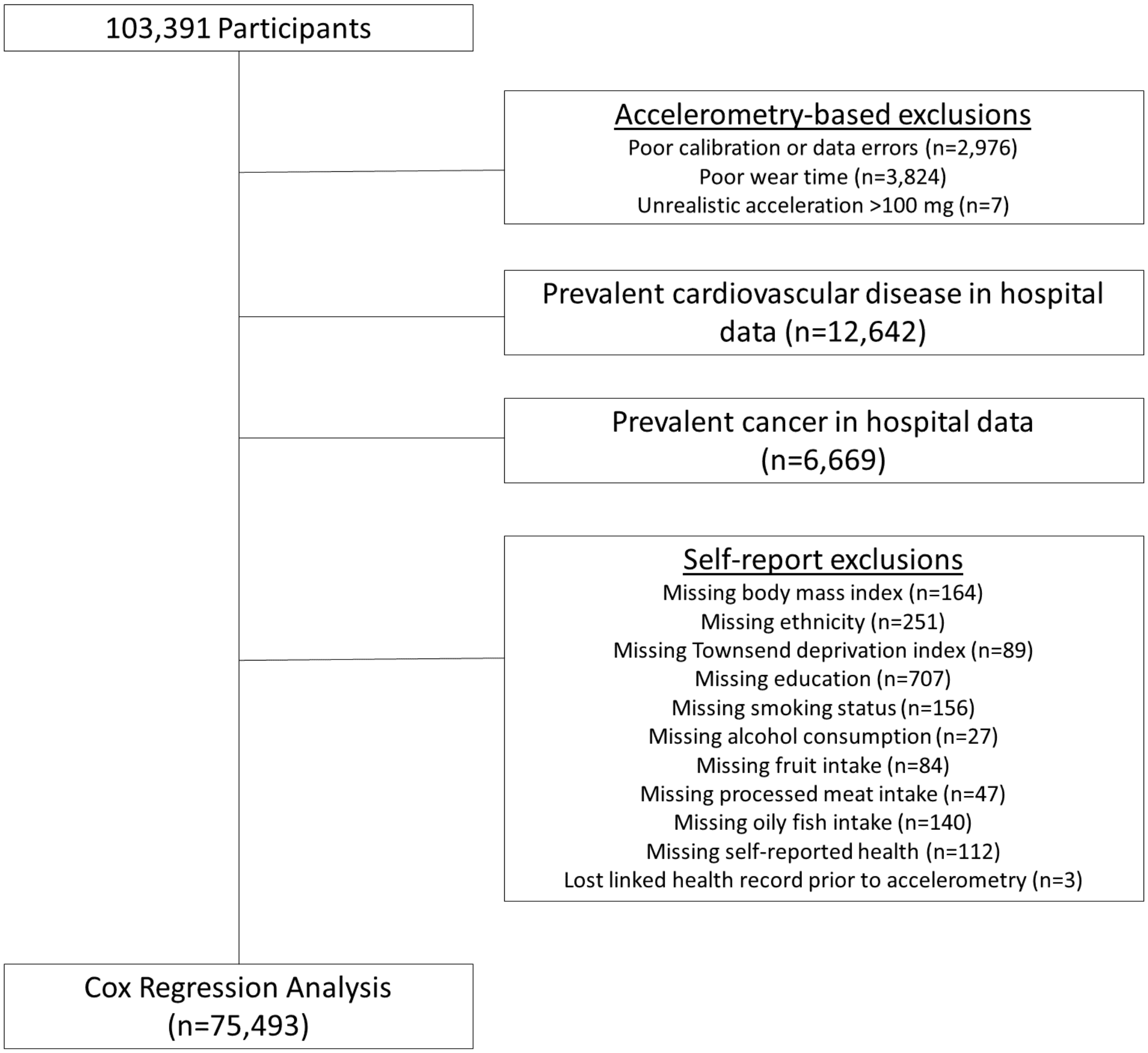

Supplemental Figure 2: Flow diagram for descriptive and survival analyses in UK Biobank physical activity cohort.

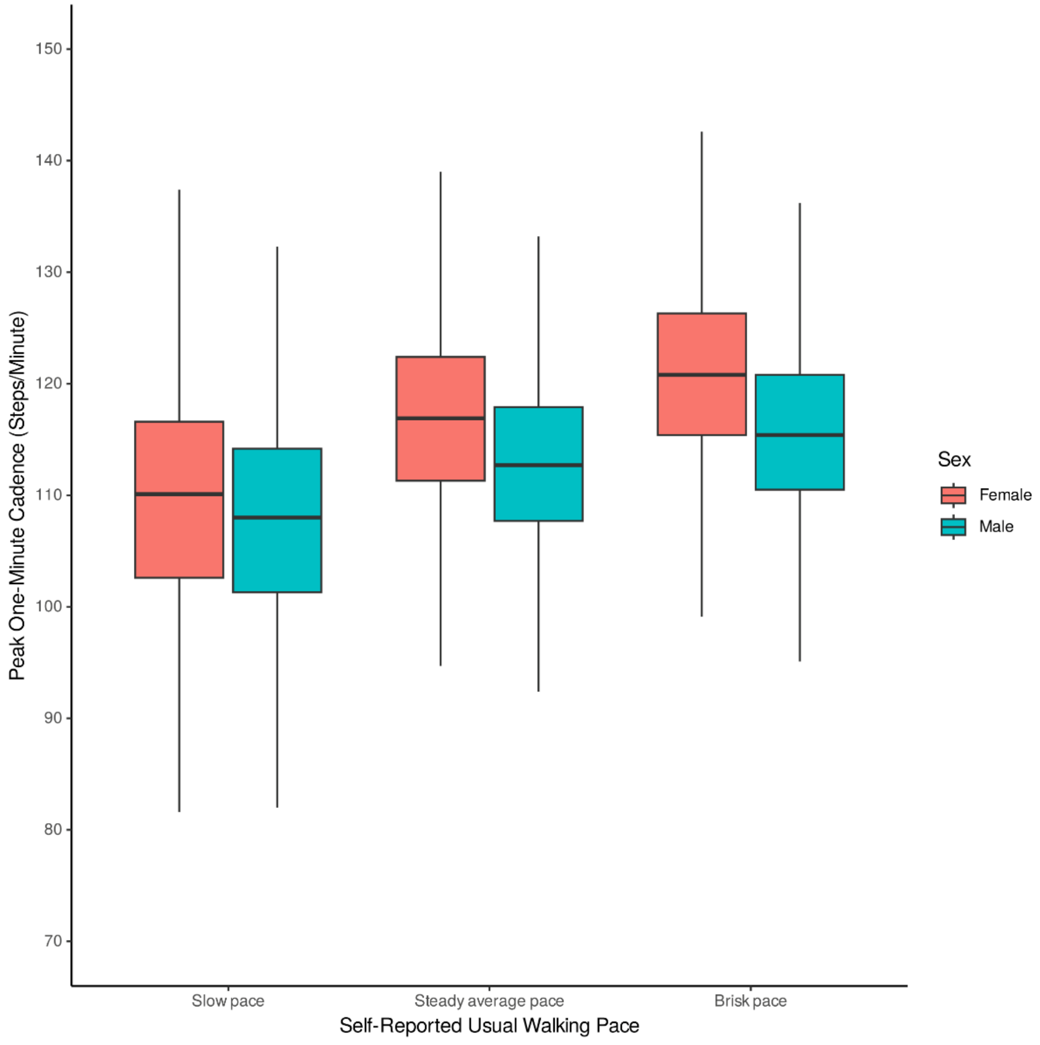

Supplemental Figure 3: Peak step cadence in the UK Biobank Physical Activity Cohort, summarised by self-reported walking pace and sex.

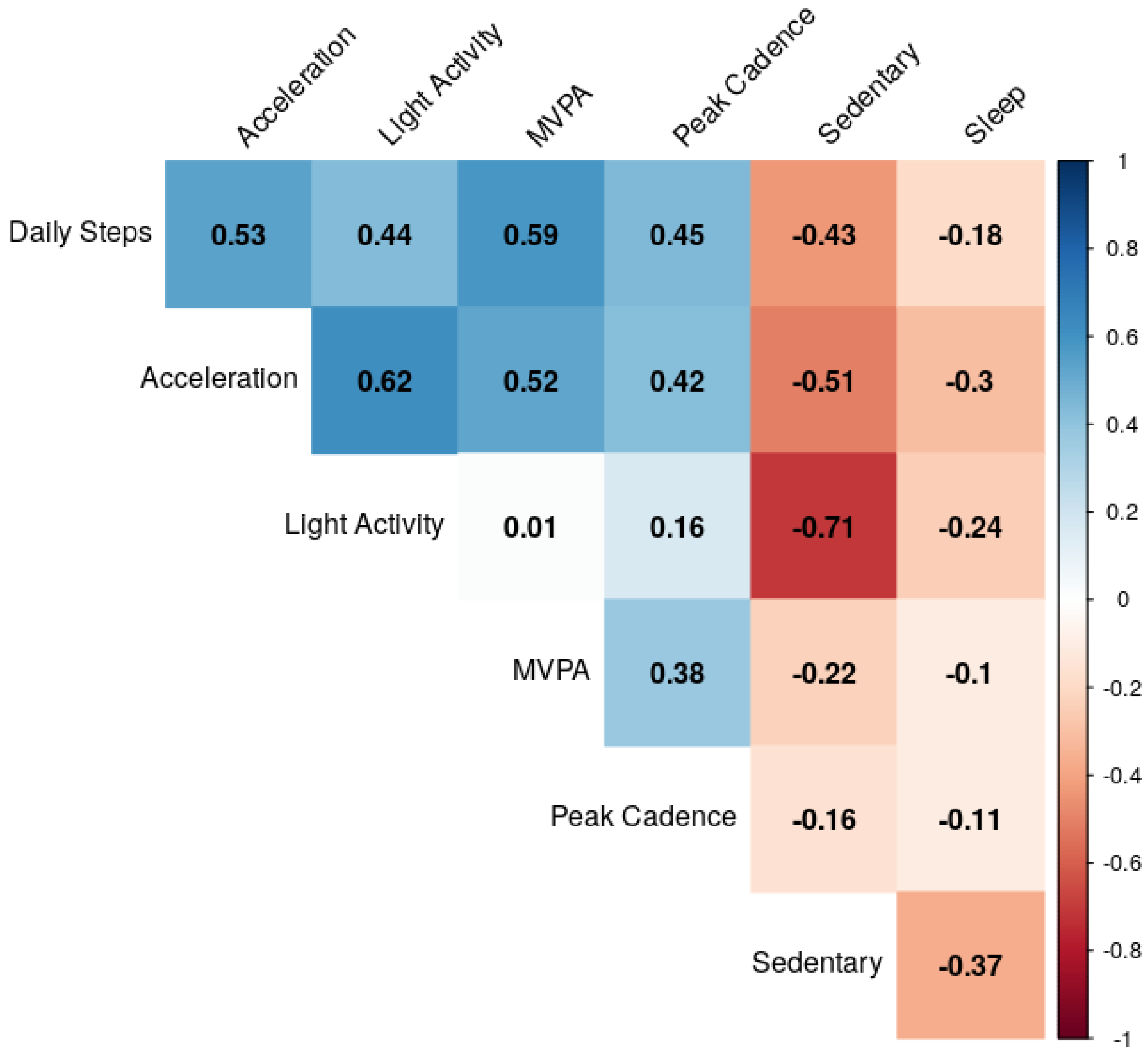

Supplemental Figure 4: Spearman's rank correlation plot between accelerometer-based physical activity metrics, including overall acceleration, daily step count, and machine learning activity classifications of time spent in sleep, sedentary behaviour, light activity, and moderate-to-vigorous activity (MVPA) in 75,493 UK Biobank participants.

Supplemental Note 1: Comparative Step Detection Models

Threshold-based step counting was conducted in R following code provided in Ducharme et al[8], wherein a single vector magnitude time series was generated for each participant with steps determined based on pre-defined acceleration thresholds following application of a 4th order Butterworth 0.25-2.5 Hz band-pass filter[8]. The Verisense algorithm, an adapted version of a smartphone stepcounting algorithm by Gu et al.[34], was applied using the R script supplied via Github repository[25].

Supplemental Note 2: Statistical Analysis, Further Detail

To establish baseline activity levels within the UK Biobank, estimated marginal means of daily step count were calculated for participants when comparing activity relative to self-reported overall health status, adjusted for age and sex using the emmeans R package[35]. Estimated marginal means of daily step counts were similarly calculated for exemplar cohorts of participants with prevalent chronic renal failure, chronic obstructive pulmonary disease, depressive disorders, and insulin dependent diabetes, as defined by ICD codes listed in the Supplemental Materials. All survival models were implemented using v.3.2.11 of R's *survival* package[36]. Floating absolute risk for each step group was calculated v.2.47 of R's *Epi* package to allow a more clear assessment of the shape of association relative to the baseline step count quintile[37,38]. This method enables comparison of HR between any step count groups without specifying an arbitrary reference group. Smoothing spline plots were generated for the association of median daily step count with all-cause mortality and cardiovascular disease mortality from terms predicted using R's *termplot* function.

Supplemental Note 3: Field and Code Usage

UK Biobank Fields

UK Biobank Data Fields: 924 – Usual Walking Pace, 23101 – Body Mass Index, 21000 – Ethnic Background, 20116 – Smoking Status, 6138 – Qualifications, 1558 – Alcohol Intake Frequency, 189 – Townsend Deprivation Index at Recruitment, 2187 – Overall Health Rating, 31 – Sex, 52 – Month of Birth, 34 – Year of Birth, 90001 - Accelerometer Data cwa format (bulk), 90010 – Start time of wear (accelerometer), 90016 – Data quality, good calibration (accelerometer), 90015 – Data quality, good wear time (accelerometer) – Derived accelerometry: 40046, Sleep – Overall Average, 40047, Sedentary- Overall Average, 40048, Light – Overall Average; 40049 , Moderate-Vigorous – Overall Average

**ICD9/10 Chronic Disease Codes (Hospital Episode Statistics)**		
	**ICD9**	**ICD10**
Any Cardiovascular Disease or Associated Death		I00-I78, I80-I99
Any Cancer or Associated Death		C00-C97
Arterial Thromboembolism	4442, 4448	I740, I741, I742, I743, I744, I745, I748, I749
Chronic Obstructive Pulmonary Disease	4929	J430, J431, J432, J438, J439, J440, J441, J448, J449
Chronic Renal Failure	5859	N180, N181, N182, N183, N184, N185, N188, N189, N19
Depressive Disorder	3119	F320, F321, F322, F323, F328, F329, F330, F331, F332, F333, F334, F338, F339
Insulin-Dependent Diabetes	25010, 25011, 25019	E100-E109, E131, E141

	ICD9	ICD10
Any Chronic Disease	01192, 01199, 0130, 01619, 01729, 01789, 0703, 1800, 1809, 1830, 1860, 1869, 1413, 1416, 1419, 1420, 1440, 1505, 1519, 1521, 1530, 1532, 1533, 1534, 1536, 1537, 1539, 1540, 1541, 1542, 1543, 1551, 1561, 1570, 1574, 1579, 1590, 1600, 1610, 1613, 1619, 1623, 1629, 1649, 1702, 1707, 1709, 1720, 1723, 1725, 1726, 1727, 1729, 1730, 1731, 1732, 1733, 1734, 1735, 1736, 1737, 1950, 1951, 1740, 1743, 1744, 1745, 1748, 1749, 1799, 1859, 1890, 1882, 1889, 1906, 1909, 1913, 1916, 1919, 1939, 28099, 2820, 2829, 2830, 2849, 2860, 2864, 2869, 2870, 2871, 2872, 28730, 28739, 2875, 1359, 24499, 2410, 2411, 2419, 2420, 2422, 2423, 2429, 25010, 25011, 25019, 25000, 25001, 25009, 25029, 2503, 2504, 2505, 25099, 2520, 2530, 25329, 2550, 25549, 2722, 27249, 2910, 2918, 2953, 2959, 2961, 3119, 3000, 3027, 3015, 3320, 3321, 3335, 3336, 33379, 3338, 3339, 3409, 3418, 3419, 34510, 34519, 34550, 34559, 3459, 3460, 3461, 3462, 3468, 3469, 4359, 3479, 3550, 3551, 3555, 3556, 3558, 35809, 3593, 3594, 3421, 3429, 34300, 3431, 3439, 3441, 3446, 3449, 3314, 3360, 3369, 3352, 4019, 4039, 3969, 3940, 3942, 3949, 4240, 4241, 4139, 4109, 4129, 4280, 4281, 4151, 4442, 4448, 4538, 4254, 4273, 4270, 4271, 4272, 4274, 4276, 4278, 4279, 4141, 4373,	A150, B180, B200, C530, C56, C620, C01, C150, C160, C170, C180, C220, C23, C250, C260, C310, C340, C300, C400, C430, C440, C450, C460, C500, C540, C61, C64, C65, C670, C680, C690, C73, C810, B500, B550, D609, D630, D66, D690, D70, D860, E011, E040, E050, E100, E110, E140, E210, E220, E230, E240, E260, E271, E780, F000, F010, F020, F100, F110, F200, F21, F220, F250, F310, F320, F400, F420, F430, F450, F500, F520, F600, F640, G060, G10, G20, G300, G35, G360, G403, G430, G450, G470, G543, G600, G700, G710, G800, G910, G950, I10, I110, I081, I340, I350, I360, I371, I200, I210, I220, I252, I251, I500, I260, I740, I820, I630, I513, I610, I64, I420, I48, I440, I711, I050, J300, J310, J320, J370, J410, J430, J450, J47, J60, J848, J850, K210, K220, K221, K224, K225, K290, K580, K350, K400, K410, K420, K430, K440, K450, K500, K510, K552, K570, K760, K811, K860, K900, M0500, M1000, M1500, M5402, M750, M8008, N180, N200, N300, N310, N40, N411, N433, N600, N61, N701, N711, N72, N800, N810, N820, N920, N970, N46, N652, H800, H900, H250, H300, H400, H490, H530

	4410, 4411, 4414, 4416, 4423, 4429, 3989, 4778, 4779, 4720, 4721, 4722, 4730, 4731, 4732, 4733, 4739, 4760, 4910, 4912, 4918, 4929, 49309, 49319, 49390, 49399, 4949, 5019, 4952, 5159, 5109, 53010, 53011, 53019, 5300, 5305, 5302, 5310, 5311, 5314, 5315, 5317, 5319, 5320, 5321, 5323, 5324, 5325, 5326, 5327, 5329, 5334, 5339, 5343, 5344, 5349, 5350, 5351, 5353, 5354, 5355, 5356, 5368, 5369, 5400, 5401, 5409, 5419, 5429, 5500, 5501, 5509, 5520, 5530, 5521, 5531, 5522, 5532, 5523, 5533, 5528, 5538, 5539, 5550, 5551, 5552, 5559, 5620, 5621, 5771, 5790, 2749, 2320, 2321, 2322, 2323, 2324, 2325, 2326, 2327, 2329, 2330, 2331, 2332, 2333, 2334, 2335, 2336, 2337, 2339, 2340, 2341, 2342, 2343, 2344, 2345, 2346, 2349, 2350, 2351, 2352, 2353, 2354, 2355, 2356, 2359, 2361, 2362, 2363, 2366, 2369, 2380, 2381, 2382, 2383, 2384, 2385, 2386, 2387, 2389, 2390, 2391, 2392, 2393, 2394, 2396, 2399, 7170, 7171, 7260, 7261, 7262, 73305, 73309, 5859, 5920, 5921, 5929, 5950, 5951, 5952, 5953, 5958, 5959, 60099, 6011, 6039, 6100, 6101, 6102, 6103, 6104, 6108, 6109, 6110, 6141, 6151, 6160, 6170, 6171, 6172, 6173, 6176, 6178, 6179, 6180, 6181, 6184, 6186, 6187, 6188, 6189, 6190, 6191, 6192, 6198, 6199, 6261, 6262, 6264, 6265, 6266, 6267, 6268, 6269, 6280, 6282, 6283, 6284, 6288,	

	6289, 6069, 3811, 3812, 3813, 3821, 3822, 3823, 3831, 3850, 3851, 3852, 3853, 3860, 3861, 3863, 3869, 3872, 3879, 3890, 3891, 3892, 3899, 3662, 3665, 3668, 3669, 3632, 3638, 3650, 3651, 3652, 3655, 3656, 3659, 3780, 3781, 3784, 3785, 3788, 3789, 3682, 3684, 3688, 3689, 3690, 3696, 3697, 3699	
